# Life in groups: the roles of oxytocin in mammalian sociality

**DOI:** 10.3389/fnbeh.2013.00185

**Published:** 2013-12-11

**Authors:** Allison M. J. Anacker, Annaliese K. Beery

**Affiliations:** ^1^Neuroscience Program, Smith CollegeNorthampton, MA, USA; ^2^Departments of Psychology and Biology, Smith CollegeNorthampton, MA, USA

**Keywords:** oxytocin, sociality, social behavior, maternal behavior, pair bond, aggression, voles, group living

## Abstract

In recent decades, scientific understanding of the many roles of oxytocin (OT) in social behavior has advanced tremendously. The focus of this research has been on maternal attachments and reproductive pair-bonds, and much less is known about the substrates of sociality outside of reproductive contexts. It is now apparent that OT influences many aspects of social behavior including recognition, trust, empathy, and other components of the behavioral repertoire of social species. This review provides a comparative perspective on the contributions of OT to life in mammalian social groups. We provide background on the functions of OT in maternal attachments and the early social environment, and give an overview of the role of OT circuitry in support of different mating systems. We then introduce peer relationships in group-living rodents as a means for studying the importance of OT in non-reproductive affiliative behaviors. We review species differences in oxytocin receptor (OTR) distributions in solitary and group-living species of South American tuco-tucos and in African mole-rats, as well as singing mice. We discuss variation in OTR levels with seasonal changes in social behavior in female meadow voles, and the effects of OT manipulations on peer huddling behavior. Finally, we discuss avenues of promise for future investigation, and relate current findings to research in humans and non-human primates. There is growing evidence that OT is involved in social selectivity, including increases in aggression toward social outgroups and decreased huddling with unfamiliar individuals, which may support existing social structures or relationships at the expense of others. OT’s effects reach beyond maternal attachment and pair bonds to play a role in affiliative behavior underlying “friendships”, organization of broad social structures, and maintenance of established social relationships with individuals or groups.

## Introduction

Across the animal kingdom, affiliative social relationships exist between individuals and their parents, offspring, mates, and non-related conspecifics. While most mammals interact prosocially only to mate or rear young, in some cases the benefits of group living have led to the evolution of complex social structures. The behaviors exhibited may vary from species to species and between individuals within a species, but the neurobiological substrates of many of these behaviors likely share common elements. The peptide oxytocin (OT) has been investigated and implicated in the context of a wide variety of social behaviors. While the majority of research on social behavior in mammals has focused on the role of OT in reproductive attachments—between a mother and her young, or between male and female mates—this review focuses on the roles of OT in mammalian social groups, and behaviors that promote group living (*sociality*).

**Figure 1:**
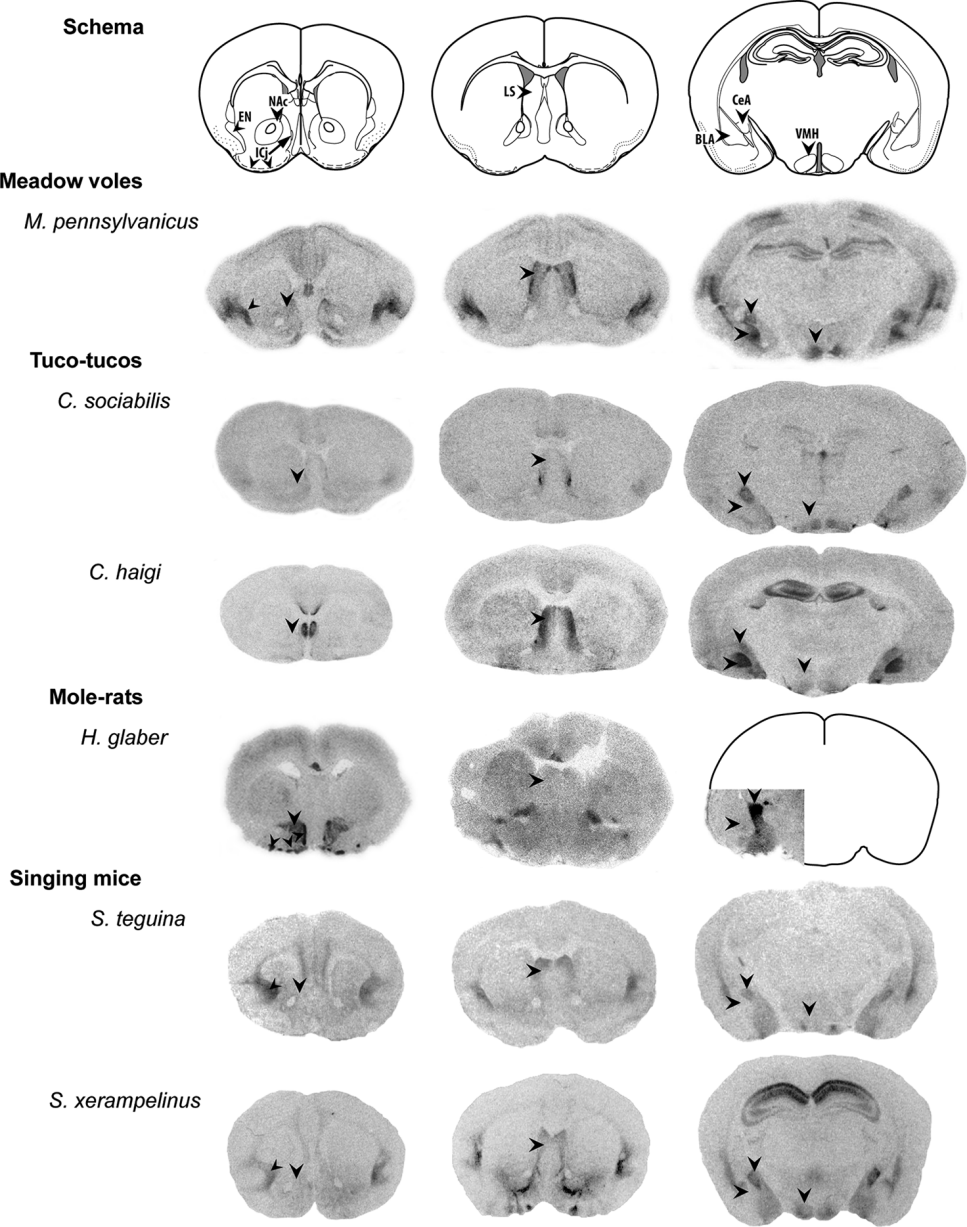
**Representative variation in OTR distribution in rodent species.** Even between closely related rodent species, the density and distribution of OTRs shows striking variation. Top row: schematic diagrams of approximate coronal sections displayed (modified from Paxinos and Franklin, 2012). Left column: labels indicate the nucleus accumbens (NAc), endopiriform nucleus (EN), and islands of Calleja (ICj). Center column: lateral septum (LS). Right column: binding in the hippocampus is seen in some animals, as well as binding in the basolateral and central nuclei of the amygdala (BLA, CeA), and ventromedial hypothalamus (VMH). Brain sections are adapted from figures of I^125^ OVTA autoradiographic assays conducted in meadow voles (Beery and Zucker, 2010), tuco-tucos (Beery et al., 2008a), naked mole-rats (Kalamatianos et al., 2010), and singing mice (Campbell et al., 2009), used with permission of original authors and publishers. Brain sizes are not to scale and image brightness and contrast have been adjusted across species to approximately match background density; comparisons of distribution of binding may be made between species, but comparisons of density should be avoided. Arrows indicate brain regions indicated in the schema.

OT is a nine-amino acid peptide which activates the oxytocin receptor (OTR) both centrally through direct neural release and in the periphery via release from the pituitary. The peptide sequence has remained highly conserved across vertebrate taxa throughout evolution. This may be due in large part to the integral role of OT in physical reproductive functions; peripheral OT release is involved the induction of labor and uterine contractions in parturition, is critical for the muscle contractions involved in milk release for nursing, and has peripheral and central effects on sexual behavior as well (reviewed in Gimpl and Fahrenholz, 2001). The broader role of OT in a variety of social behaviors and related processes may derive from its central role in these reproductive behaviors. While the importance of OT for social functions appears nearly universal, central OTR distribution varies between species and may relate to species-typical social behavior (Insel and Young, 2000; Donaldson and Young, 2008; Figure 1).

Many social behaviors share common features, but what it means to be social may be different for different species (Goodson, 2013). For example, in farm animals it means having a passive tolerance for being surrounded by conspecifics without demonstrating aggression (Estevez et al., 2007), which may be most similar to the mechanisms supporting large aggregations of animals in the wild. In contrast, specific social relationships in some primate species may involve actively engaging with and grooming certain individuals to form bonds or use as currency (Henzi and Barrett, 1999). Large social groups may rely on both specific and non-specific social relationships; for example, in geladas functional social groups are a subset of much larger aggregations (Bergman, 2010). In order to understand the mechanisms supporting naturally occurring mammalian groups, we focus on the roles of OT in support of specific social structures for particular mammalian species. We first review the roles of OT in social behaviors where they have been best studied: maternal affiliation and pair-bonding in prairie voles. We then move on to what is known about the regulation of OT in a variety of mammalian species that spend their lives in social groups. This comparative perspective on sociality will ultimately shed light on common elements of regulation in the OT system, as well as the specific and varied behaviors that permit sociality—including, but not limited to OT-related behaviors.

## Maternal behavior and family groups

For most mammals, early life is experienced in a group because of the obligatory role of the mother in nursing and caring for young. Even in species in which the mother’s time nursing is brief, such as rabbits, the early environment is often shared with siblings. Little is known about the role of OT in social behavior among juvenile mammals, but in many species, OT plays a key role in the regulation of maternal behavior, along with other hormones and neurotransmitters (see Bosch and Neumann, 2012; for a recent review).

Recognition is essential for maternal behavior in certain species or at particular phases in development. For sheep, mothers must learn to recognize their own offspring within a few hours of giving birth, as the lamb begins to walk; OT is critical for this attachment (Kendrick et al., 1997). Fifteen-day old rat pups also learn to recognize the scent of their mother, in a process which requires activation of the OTRs, as it is blocked with intra-cerebroventricular administration of a receptor antagonist (Nelson and Panksepp, 1996). There is evidence that OT, specifically acting on receptors in the medial amygdala, is essential for general social recognition in mice (Ferguson et al., 2000, 2001), and OT acting in the olfactory bulb maintains social memory in rats (Dluzen et al., 2000). Further, endogenous OT alters the natural social tendencies of rats and mice, as central administration of an OTR antagonist decreases social investigatory behavior (Lukas et al., 2011).

OT facilitates initiation of maternal behavior, as has been demonstrated by lesion studies and receptor antagonist administration in rats and genetic knockout studies of the OTR in mice (reviewed in Campbell, 2008; Bosch and Neumann, 2012). In addition, administration of OT centrally can induce maternal behaviors in naïve juvenile female rats (Pedersen and Prange, 1979).

Although research on the development of paternal behavior is sparse, there is some evidence for a role of OT in male parenting and alloparenting. Juvenile and adult male prairie voles have a transient increase in OT when exposed to pups (Kenkel et al., 2012). While OT administration does not increase the already high levels of parental behavior in prairie voles, an OTR antagonist (along with a vasopressin receptor antagonist) decreases spontaneous parental behavior, indicating that OT does play a role (Bales et al., 2004). Alloparental behavior among naïve males and females is important in many mammalian species where older siblings or others in the community help rear young, and these communal nests or groups serve as the basic social structure (for review, see Hayes, 2000). Alloparental behavior in juvenile prairie voles is positively correlated with OTR levels in the NAc and caudate-putamen, and negatively correlated with receptor levels in the LS (Olazabal and Young, 2006). A corresponding relationship is seen across species: less spontaneously parental mice and meadow voles have higher receptor levels in the LS and lower levels in the NAc and caudate-putamen than more parental rats and prairie voles (Olazabal and Young, 2006).

OT also plays a role in continued maternal care in rats, such as licking and grooming and arch-backed nursing of young. Central administration of an OTR antagonist decreases levels of licking and grooming in mothers that exhibited a high degree of this type of care (Champagne et al., 2001). Interestingly, OTR levels in several brain regions differ between high- and low-licking and grooming mothers; OTR levels were higher in the bed nucleus of the stria terminalis, central nucleus of the amygdala, ventral LS, medial preoptic area, and paraventricular nucleus of the hypothalamus of the high-licking and grooming mothers (Champagne et al., 2001).

Together with the onset of maternal behaviors such as nest-building and pup retrieval, maternal aggression toward intruder rats appears to involve OT, as has been demonstrated in many studies, although the precise timing and mechanisms are still unclear (Campbell, 2008; Bosch and Neumann, 2012). In addition to protecting young, aggression is a key instrument in determining social relationships and hierarchies. OT is central to multiple aspects of parent-offspring attachments, and for mammals, these relationships form the basis of the first group-living environment experienced by young.

## Monogamy and Mate-Pairs

While cohabiting mate-pairs do not necessarily constitute a group in the sense it is usually considered, they may inform us about the mechanisms that contribute to group living, and the mechanisms supporting pair-wise social bonds are well studied. Monogamy has evolved independently in a variety of taxa. While it is common among birds, occurring in around 90% of avian species, it is rare among mammals, occurring in as few as 3% of species (Kleiman, 1977). Most monogamous species are socially but not genetically monogamous, indicating that they choose to spend their time with one individual with whom they have a pair bond, although extra-pair copulations may occur (e.g., Solomon et al., 2004; Ophir et al., 2008).

Prairie voles are undoubtedly the species for which the neurobiological mechanisms underlying pair bond formation have been best characterized. In conjunction with investigations of prairie voles, a number of studies have compared closely related vole species’ behavior and neurobiology (reviewed in Young et al., 2008). Such comparative studies have examined the expression and distribution of the OT peptide and its receptor. Distribution of OTRs differs between species of voles that vary in mating system, with distinct and almost non-overlapping limbic regions containing dense receptor expression (Insel and Shapiro, 1992). Some particularly interesting conclusions have been made based on comparison of monogamous species (*Microtus ochrogaster* and *M. pinetorum*, prairie and pine voles) and non-monogamous species (*M. pennsylvanicus* and *M. montanus*, meadow and montane voles), although this remains a small sample size within one particular taxonomic branch. Monogamous prairie and pine voles exhibit significantly lower levels of OTRs in the LS compared to non-monogamous montane and meadow voles. Other regions do not show consistent patterns of differences in expression level between monogamous and non-monogamous vole species. For example, although OTR level is significantly higher in the NAc and bed nucleus of the stria terminalis of prairie voles compared to montane voles, there is no significant difference in these regions when pine voles are compared to meadow voles. The expression of OT itself is quite similar among vole species, indicating that changes in the receptor expression and distribution are more likely responsible for the evolution of relevant differences in social behavior (Wang et al., 1996).

Another comparative study on nonapeptide receptor distribution and mating system examined two species in the genus *Peromyscus*: the monogamous California mouse (*P. californicus*) and the polygamous deer mouse (*P. maniculatus*) (Insel et al., 1991). Differential levels of OTRs are observed in several brain regions, including the LS. However, the direction of the difference is different in the *Peromyscus* species compared to *Microtus*: the monogamous *P. californicus* had greater levels of OTR binding in the LS than did the polygamous *P. maniculatus*. While these results may not support the hypothesis that the level of OTRs corresponds with the monogamous or promiscuous mating systems, consistent with the idea that there may be important differences in OTR distribution that are relevant for social organization (Insel et al., 1991). In order to understand the significance of these differences it will be important to examine the role of the receptors in each region as they relate to each species’ specific social behaviors.

The functional significance of species differences in OTR expression patterns has been demonstrated by studies of pair bond formation in voles. Partner preference formation in female prairie voles is facilitated by infusion of OT into the NAc (Liu and Wang, 2003) as well as by overexpression of the OTR in the NAc using adeno-associated virus (Ross et al., 2009). It is likely that OT in the NAc is particularly important for the rewarding aspects of social attachments: recent evidence indicates that social reward conditioning in mice requires activation of presynaptic OTRs and postsynaptic serotonin 5-HT1B receptors in the NAc, indicating that this mechanism is not unique to social monogamy in prairie voles (Dolen et al., 2013). Not all affiliation relies on NAc OT, however. Overexpression of the OTRs in female meadow voles, which typically show very little OTR expression in the NAc (Insel and Shapiro, 1992), is not sufficient to induce a partner preference or increase the amount of total time spent in proximity of both stimulus animals. Other social species, such as social tuco-tucos (described below) have no detectable OTR in the NAc. These and other studies indicate that while OTR activation in the NAc is necessary for male-female attachments in female prairie voles (Young et al., 2001; Liu and Wang, 2003), it is not sufficient to induce such attachments in other species; there must be other circuitry that further determines how actions of the OT system affect such relationships.

Within-species variations in social behavior have also revealed a role for the OT system in monogamous behaviors. Male prairie voles that become paired in a semi-natural setting have a higher density of OTRs in the NAc than those that remain single (Ophir et al., 2012), while paired and single females do not exhibit differences in receptor levels except those dependent on pregnancy status (Zheng et al., 2013). Future studies focusing on such individual variability and correlates in the social decision making network will further elucidate the mechanisms that contribute to group-living.

The role of OT in prosocial behavior has also been studied in monogamous primates via manipulations of the OT system. Monogamous marmosets given OT increased social behavior such as initiation of huddling with a partner during cohabitation or decreased the latency to approach the partner during a partner preference test after three weeks of cohabitation; an OT antagonist decreased social behaviors such as initiation of proximity or food sharing with a partner during cohabitation (Smith et al., 2010).

Recent evidence has shown that OT or OT-like peptides are important for the social bond formation between mates in non-mammalian species as well. Zebra finches require activation of OT-like receptors in order to demonstrate preference for a pair-mate (Klatt and Goodson, 2013), and an antagonist decreased bonding (Pedersen and Tomaszycki, 2012), as has been demonstrated in prairie voles. Interestingly, there is evidence that OT is more vital for the development of the social preference in females than in males. Monogamous cichlid fish also regulate social affiliation during bond formation via activation of OT- and vasopressin-like pathways, although the bond formation itself is not affected by manipulation of these pathways (Oldfield and Hofmann, 2011). In each of these species, it is clear that OT plays a central role in the formation of pair bonds between mates—an integral feature of the monogamous mating system.

## Mechanisms supporting group-living

While the vast majority of mammalian neuroscience research is conducted on rats and mice (Beery and Zucker, 2011), interest in the mechanisms supporting specific social behaviors has necessitated examination of less commonly researched species. Among mammals, investigation of the link between OT circuitry and sociality has begun in a variety of species that live in groups, including African mole-rats, South American tuco-tucos, meadow voles, singing mice, striped mice, and multiple primate species. These studies make use of variation in group-living behavior across species, seasons, and social contexts. A summary of findings concerning specific relationships between OT and social structure in rodents appears in Table 1; we explore those models finding social variation related to OT circuitry in greater detail below. In spite of the variation in OT’s roles in social behaviors and distribution of the receptor across species, the expression of the peptide itself is largely conserved across vertebrate taxa, from production in the hypothalamus to distribution in the forebrain (Insel and Young, 2000). OTR distribution is much more variable; Figure 1 illustrates this variation across several rodent taxa, including some closely related species. Gathering additional neurochemical data on diverse species with variation in sociality will allow us to bridge ecological and behavioral research on social behavior with neurobiology to synthesize key findings (O’Connell and Hofmann, 2011).

**Table 1:** **Rodent species for which OT and group-living behaviors have been examined**.

**Rodent species or taxonomic group**	**Phenotypes of interest for studying group living**	**Studies of oxytocin circuitry**	**References**
Meadow voles *Microtus pennsylvanicus*	Seasonal variation in social behavior (territorial vs. communal), inducible by changing laboratory light cycles	OTR distribution across seasonal social phenotypes	Parker et al. (2001), (Beery and Zucker 2010)
				Effects of OT and antagonist infusions on same-sex partner preferences	Beery and Zucker (2010)
Tuco-tucos Genus *Ctenomys*	Species-rich genus with multiple solitary and social species; additional variation in social behavior within the family (Octadontidae)	OTR distribution in *C. sociabilis* and *C. haigi*	Beery et al. (2008a)
Mole-rats Family Bathyergidae	The only eusocial rodents; solitary and extremely social species within the family, as well as variation between breeders (e.g., queen) and subordinates	OT-neurophysin fiber distribution in naked mole-rats (*Heterocephalus glaber*)	Rosen et al. (2008)
		OTR distribution and OT-neurophysin fiber density in naked mole-rats and cape mole-rats (*Georychus capensis*)	Kalamatianos et al. (2010)
		OT neuron number and social status (dominant breeder or subordinate non-breeder)	Mooney and Holmes (2013)
Singing mice Genus *Scotinomys*	Multiple social species with different social structures	OTR distribution in *S. teguina* and *S. xerampelinus*	Campbell et al. (2009)
Striped mice *Rhabdomys pumilio*	Philopatric (group-living) males and dispersers	OT immunoreactivity in group/solitary laboratory housing and induced breeding status (no differences)	Schradin et al. (2013)

Complete references can be found in the References section below. OT: oxytocin; OTR: oxytocin receptor.

### Meadow Voles

Microtine rodents (voles) exhibit a high degree of behavioral diversity, from monogamy to promiscuity, and from territorial aggression to cohabitation and cooperative breeding. Meadow voles (*Microtus pennsylvanicus*) exhibit the latter dichotomy within a single species under changing environmental conditions. Meadow voles have been extensively studied as a non-monogamous counterpart to prairie voles but while they are often described as “asocial”, this solitary behavior is limited to summer months. Female meadow voles are solitary and aggressive during the breeding season, during which time they maintain non-overlapping territories (Madison, 1980; Webster and Brooks, 1981). In winter months, meadow voles remain active, and as the season progresses they share space and form communal nests of 2–10 individuals (Madison et al., 1984). This seasonal shift in social behavior can be triggered with day length manipulations alone: both male and female meadow voles form selective attachments with same-sex individuals during short, winter-like day lengths in the laboratory (Parker and Lee, 2003; Beery et al., 2008b, 2009; Beery and Zucker, 2010). Male meadow voles exhibit less pronounced seasonal variation in the field as well as in the laboratory (Boonstra et al., 1987, 1993; Beery et al., 2009). Because meadow voles form same-sex partner preferences but are not monogamous, they provide a model for understanding affiliation outside the context of reproduction. Multiple studies have explored the role of OT in this peer affiliation.

Centrally administered OT enhances partner preferences in female meadow voles, indicating that activation of the receptor plays a role in the preference for familiar individuals. However, blockade of the OTR does not block preferences, suggesting that OT is not necessary for baseline preference formation, and that other mechanisms also play a role (Beery and Zucker, 2010). Unlike in prairie voles, blockade of dopamine receptors with haloperidol does not interfere with formation of partner preferences in meadow voles (Beery and Zucker, 2010). Together with the lack of impact of overexpression of OTRs in the NAc of female meadow voles (Ross et al., 2009, described above), this suggests that the circuitry underlying non-reproductive preferences does not include the same NAc oxytocin/dopamine actions involved in the monogamous bonds formed in prairie voles.

Alternative potentially important oxytocinergic pathways have been identified by receptor autoradiography. OTR densities in female meadow voles vary with day length and social behavior in multiple brain regions including the LS and central nucleus of the amygdala (Parker et al., 2001; Beery and Zucker, 2010). The LS in particular holds promise as a potential region of interest; OTR density in the LS is significantly and negatively correlated with time spent huddling in the partner preference test (Beery and Zucker, 2010). This within-species correlation parallels between-species findings, where prairie voles had lower OTR expression in the LS (Insel and Shapiro, 1992) and higher levels of huddling compared with meadow voles (i.e., Lim et al., 2004), as well as findings in tuco-tucos (detailed below).

On first consideration it may seem counterintuitive that OT administration enhances partner preferences but greater OTR level in the LS is associated with decreased huddling time. While OT is best known for enhancing prosocial behaviors, increasingly studies are finding that these impacts are context and circuit specific, and that OT signaling in the LS in particular may contribute to agonistic behaviors or social avoidance. Endogenous OT release in the LS during social defeat stress leads to enhancement of the conditioned fear response in mice, an effect which can be increased by overexpression of the OTR in the septum, but not by exogenous OT administration (Guzman et al., 2013). Exogenous OT administrated to the LS enhances social recognition (although blockade of the OTRs does not block recognition) (reviewed in Gabor et al., 2012), and may serve to decrease tolerance for unknown individuals, similar to ingroup-outgroup dynamics in human studies, discussed below. Partner preference formation in prairie voles is accompanied by an increase in aggression toward unfamiliar individuals (Getz et al., 1981). OT may play an important role in both “approach” and “avoid” aspects of social contact.

At present, these studies demonstrate that the behavioral changes that are central to the seasonally-changing social structure of meadow voles are associated with changes in OTR levels and affected by administration of OT, indicating that the OT system is involved in species-specific social systems. Further confirmation of the role of specific brain regions and oxytocinergic mechanisms in the seasonal shift in social behavior will increase understanding of how these behaviors are shaped naturally, and how changes may be induced.

### Tuco-Tucos

The relation between OTR distribution and group-living is also being explored in the genus *Ctenomys* (tuco-tucos)**, which consists of over 50 species of South American burrowing rodents. All tuco-tucos for which behavioral descriptions exist are promiscuous breeders. The majority of species are solitary, but select species including *C. sociabilis*, *C. peruanus*, and *C. opimus* are social, as are the Octodontidae family: degus (*Octadon degus*) and coruros (*Spalacopus cyanus*).

Initial neurochemical comparisons have focused on the social tuco-tuco (*C. sociabilis*) and the solitary Patagonian tuco-tuco (*C. haigi*). Social tuco-tucos live in groups of up to six females, sometimes accompanied by a male (Lacey et al., 1997; Lacey and Wieczorek, 2004). In contrast, adult Patagonian tuco-tucos do not share burrow systems (Lacey et al., 1998). These species exhibit dramatic variation in OTR distribution and density. Colonial tuco-tucos have higher levels of OTR in the central nucleus of the amygdala than do solitary *C. haigi*, and strikingly lower levels in the LS (Beery et al., 2008a). These species differences are similar in direction to the differences between monogamous and promiscuous vole species, and to the within-species social behavior differences in meadow voles described in the previous section. Combined evidence from these species suggests that the LS may be a region of particular importance for regulation of social behavior. Role the LS in social behaviors has been repeatedly identified as important (O’Connell and Hofmann, 2011). While LS OTR levels are greater in less social species or individuals in all of the examples mentioned above, OT may yet play a different role in the LS of different species (Insel and Shapiro, 1992). For example, lesions of the LS decrease social behavior in one subspecies of deer mice and increase it for another more social subspecies (MacDougall et al., 1975). Further study of this anatomical region will reveal how these similarities and differences are related to one another.

Unlike monogamous voles and naked mole-rats, neither tuco-tuco species exhibits notable OTR binding in the NAc (Beery et al., 2008a; Figure 1), suggesting that OT reception in this brain region is not a critical component of group living in *C. sociabilis* and that other mechanisms may support this behavior. While tuco-tucos are evolutionarily distant from new world rodents, they are members of the Histrichognathi suborder of Histrichomorph rodents along with African mole-rats. Broader phylogenetic comparisons of OTR binding will aid in determining how OTR distribution varies with both evolutionary history and social behavior; comparisons of seven tuco-tuco species, degus, and coruros are currently underway (Beery and Lacey, pers. comm.).

### Mole-Rats

The Bathyergidae family of African mole-rats contains classically solitary species as well as the only known mammalian examples of eusociality (a social system in which a colony of animals—often with static castes—exhibits cooperative breeding with only a few members participating in reproduction). The naked mole-rat (*Heterocephalus glaber*) is the most social of these species. They live in highly cooperative groups of 70–80 (and up to 300) individuals, most of which are reproductively suppressed non-breeders (Sherman et al., 1991). Non-breeders of both sexes provide alloparental care to the pups (Jarvis, 1981; Lacey and Sherman, 1991). Naked mole-rats have been anatomically and histochemically compared to solitary cape mole-rats, and also compared within species across breeding status (Table 1, and findings below).

Studies of oxytocin-neurophysin immunoreactive processes in mole-rats have demonstrated a preponderance of OT fibers in the NAc (Kalamatianos et al., 2010). Naked mole-rats have a greater density of these fibers in the NAc relative to cape mole-rats, with a similar difference in the septum but to a lesser extent (Kalamatianos et al., 2010). OTR densities in these species have also been characterized, with higher receptor density in eusocial naked mole-rats relative to solitary cape mole-rats in the NAc as well as the indusium griseum, nuclei of the amygdala, bed nucleus of the stria terminalis, and hippocampal CA1 region (Kalamatianos et al., 2010). Naked mole-rats have a surprising lack of vasopressin-immunoreactive fibers in the LS (Rosen et al., 2008), as might be expected if vasopressin is associated with mediation of agonistic interactions. While vasopressin and the vasopressin type 1a receptor are thought to be most important for male social behavior (Carter, 2007), a few studies in other mammalian species suggest that vasopressin neurotransmission may also play a role in female behavior (Caldwell and Albers, 2004; Rosen et al., 2006, 2007).

Naked mole-rats also exhibit within-species variation in the OT system that is dependent on breeding status. Subordinate non-breeding mole-rats of both sexes have significantly higher numbers of oxytocin-immunoreactive cells in the paraventricular nucleus of the hypothalamus when compared to breeders, or to subordinates separated from the colony and placed in male-female pairs. Intriguingly, these differences appear to be independent of sex and mating (Mooney and Holmes, 2013). Future studies to more thoroughly explore the OT system (including receptor changes with social status in naked mole-rats) will enhance what this unique family adds to what is known about OT and sociality.

### Singing mice

Two species of singing mice, *Scotinomys teguina* and *S. xerampelinus*, both exhibit remarkable vocal communication and are considered social animals. However, they differ greatly in their social structure as well as in distribution of OTRs. *S. teguina* exhibits signs of greater maternal investment in offspring, and less dense space use compared to *S. xerampelinus*. This may be related to greater OTR levels in brain regions associated with sociospatial memory in the more densely living *S. xerampelinus*, especially in the hippocampus and medial amygdala, which may aid orientation toward particular resources. *S. xerampelinus* also exhibits higher receptor levels in the shell of the NAc and the central nucleus of the amygdala, both important for maternal behavior (Campbell et al., 2009; Figure 1). While additional studies are needed to determine causality, these results further support a role for OT in behaviors that govern social structure.

### Human and non-human primates

Social structure is maintained in group-living species by many behaviors, from cooperation to aggression. In primates, increased complexity of social systems corresponds with increased neocortical volume (Dunbar, 1998), and many cognitive factors influence social decision making (Carter et al., 1997). Nonetheless, OT both influences and responds to social behavior in these species.

In many non-human primates, relationships are maintained with allogrooming. Allogrooming in chimpanzee dyads with a close social relationship produces a greater increase in OT levels than grooming between less close individuals (Crockford et al., 2013). Among rhesus macaques, there is a positive correlation between social rank and amount of grooming received (Schino, 2001). These kinds of interactions may serve to create and strengthen affiliative alliances between individuals, which can contribute to the structures within social groups.

Behavioral and fMRI studies in humans have demonstrated that intranasal OT administration affects many aspects of social interactions as well as perceptions of social situations (reviewed in Zink and Meyer-Lindenberg, 2012). For example, OT increases trust (Kosfeld et al., 2005), eye gaze (Guastella et al., 2008), and the ability to infer another’s emotional state (Domes et al., 2007). It decreases the reaction to some social stimuli (Kirsch et al., 2005), particularly negative or aversive stimuli. One study examining the cross-generational effects of parenting indicated that both positive parenting behaviors and peripheral OT levels correlated with positive features of interactions between friends in young children (Feldman, 2012). These effects of OT on specific social interactions can shed light on the mechanisms that broader social structure depends upon.

### Other Vertebrates

Although the involvement of oxytocin-related nonapeptides in sociality in non-mammalian species is largely beyond the scope of this review, there is a growing literature in this domain (for reviews, see O’Connell and Hofmann, 2011; Goodson, 2013). Using birds as one example, gregarious and flocking species (*Taeniopygia guttata*, *Lonchura punctulata*, and* Uraeginthus angolensis*) have higher levels of oxytocin-like binding sites in the LS compared to those that are more territorial (*Pytilia melba* and *Uraeginthus granatina*) (Goodson et al., 2009). Further, in birds that change their flocking behavior seasonally (*Spizella pusilla*), mesotocin innervation of the LS increases in flocking months (Goodson et al., 2012). This illustrates that oxytocin-like systems are relevant for group social structures in other vertebrates as in mammals.

## Non-Affiliative behaviors that support social structure

Pro-social behaviors are fundamental building blocks of group living, however these are not the only behaviors necessary for the foundation and maintenance of a social structure. Agonistic social behaviors such as aggression or exclusion of individuals from a group are also vital for aspects of group living such as establishing social hierarchies, and for maintaining group identity or territory in the face of outsiders. There is increasing evidence that OT plays a role in these behaviors in addition to the pro-social behaviors described previously.

OT is involved in both acute aggressive interactions and lasting dominance relationships. The peptide is released in the LS during an acute social defeat in rats (Ebner et al., 2000), which can have lasting effects because initial interactions can be remembered for long periods of time, and thus contribute to a stable social dominance hierarchy (Adkins-Regan, 2005). In established hierarchies, something different is observed. Dominant female rhesus macaques have higher levels of serum OT than subordinates (Michopoulos et al., 2011, 2012), and dominant male squirrel monkeys exhibit greater levels of aggression when given OT infusions (Winslow and Insel, 1991). Conversely, subordinate male rats decrease OTR expression in the long-term establishment of dominance roles (Timmer et al., 2011). Dominant male cichlid fish have higher levels of isotocin in the hindbrain (Almeida et al., 2012), and aggressive dominant three-spined sticklebacks have higher levels of isotocin in the brain as well (Kleszczynska et al., 2012).

OT also contributes to anti-social and agonistic behaviors among humans. Subjects experiencing monetary loss in a laboratory game relative to other (simulated) participants report greater levels of envy and gloating when given intranasal OT, compared to those given a placebo (Shamay-Tsoory et al., 2009). Individuals with borderline personality disorder show *decreased* trust and cooperation when given OT, compared to placebo, while control participants do not show any significant effects of OT on their responses (Bartz et al., 2011). Several other studies have also demonstrated the importance of individual context in mediating the effects of OT. One showed that OT improves the recollections of maternal care received in more securely attached men, while it worsens recollections in more anxiously attached men (Bartz et al., 2010). Another demonstrated that OT increases the distance men in relationships put between themselves and an attractive female, but does not have the same effect in single men (Scheele et al., 2012). Some negative effects of OT on social behaviors may be due to the peptide’s ability to produce anxiogenic effects, such as increasing startle response and memory of negative stimuli (Striepens et al., 2012; Grillon et al., 2013). For example, participants in a prisoner’s dilemma game making financial decisions on behalf of a group report greater protection and trust of their own in-group when threatened with a non-cooperative out-group, and accordingly make more decisions that would benefit the in-group and punish the out-group when administered OT (De Dreu et al., 2010).

These findings on OT’s role in aggression, dominance, trust, and negative emotions toward others indicate that the OT system is relevant for maintaining group-living not only by influencing pro-social behavior, but also by affecting agonistic social behaviors that can solidify group cohesion and protect against others. Discovery of these processes in comparative studies has translational value such as producing targets to treat disrupted social behavior, a primary component of many psychiatric disorders, in addition to providing a greater appreciation of the diverse mechanisms of sociality.

## Conclusions

OT is involved in sociality at multiple levels, from the support of individual behaviors to associations with specific social structures. In mammals, OT is integral to the development of parental care, recognition, and attachment of mother and offspring. It is also involved in sexual behaviors and in some species the monogamous pair bond. Comparative studies allow for “natural experiments” regarding the roles of OT in animal behavior. Recent studies in group-living meadow voles, tuco-tucos, mole-rats, and singing mice have demonstrated the evolutionary lability of the OTR system, and are helping to identify species-specific strategies for social living. The LS and amygdala have repeatedly been revealed as important neuroanatomical loci for affecting specific social behaviors and social living strategies. These regions may be particularly important for modulating social anxiety and territoriality or aggression, which in turn regulate what kind of group-living can develop. By targeting natural variation in group-living behavior across a variety of mammalian and non-mammalian species we will gain a much better understanding of the mechanisms—oxytocinergic and otherwise—that promote life in groups.

## Conflict of interest statement

The authors declare that the research was conducted in the absence of any commercial or financial relationships that could be construed as a potential conflict of interest.
